# CCK-1R-selective and non-selective cholecystokinin antagonists, lorglumide, and proglumide increased toxicity of carboplatin to granulocyte–macrophage progenitor cells (CFU-GM) of bone marrow of rats

**DOI:** 10.1007/s00210-025-04391-6

**Published:** 2025-06-30

**Authors:** Beáta Pelles-Taskó, Angelika Varga, Krisztina Géresi, Béla Juhász, Zoltán Szilvássy, Ilona Benkő

**Affiliations:** 1 Department of Pharmacology and Pharmacotherapy, Faculty of Medicine, University of Debrecen, Nagyerdei Blv. 98, 4032 Debrecen, Hungary; 2 Department of Anatomy, Histology and Embriology, Faculty of Medicine, University of Debrecen, Nagyerdei Blv. 98, 4032 Debrecen, Hungary; 3 Laboratory of Clinical Immunology, Medical Center, Hungarian Defense Forces, 1134 Budapest, Hungary

**Keywords:** Lorglumide, Proglumide, Cholecystokinin receptors, Granulocyte–macrophage progenitor (CFU-GM), Myelotoxicity, Carboplatin

## Abstract

Cholecystokinin antagonists are investigated to use against pancreas and hepatocarcinomas, the risks of which are higher in obesity with poorer prognosis than in nonobese patients. We studied their effects on granulocyte–macrophage progenitor (CFU-GM), the key target of myelotoxicity of chemotherapy. Colony formation of CFU-GM was studied after the same molar doses of proglumide or lorglumide (iv, 5 days). Direct toxicity of carboplatin was determined against CFU-GM progenitors of LETO rats pre-treated with proglumide or lorglumide and against progenitors of their obese counterparts OLETF rats. Cholecystokinin receptors were studied by qPCR. Proglumide and lorglumide damaged granulopoiesis *in vivo* and inhibited CFU-GM of LETO rats dose-dependently *in vitro*. The CCK-1R-selective lorglumide caused more powerful inhibition than non-selective proglumide both *in vitro* and *in vivo*. Increased carboplatin toxicity was measured *in vitro* against CFU-GM obtained from either proglumide or lorglumide pre-treated rats. Carboplatin toxicity was significantly higher after lorglumide than proglumide pre-treatment, which confirmed protective effects via CCK-1R. Carboplatin damage was higher on CFU-GM progenitors of OLETF rats with CCK-1R deficiency than that of LETO rats. We detected both CCK-1R and CCK-2R in progenitors of bone marrow. Gene expressions of both CCK-Rs decreased after proglumide administration. Cholecystokinin antagonists affected granulopoiesis and sensitized granulocyte–macrophage progenitors against carboplatin toxicity presumably by inhibition of the protective role of cholecystokinin via CCK-1R. It is the first proof about the presence and possible role of CCK-1 receptor in granulopoiesis. These might have value if CCK antagonists are used in malignancies, obesity, or with immunosuppressive therapies.

## Introduction

Recently, more and more functions of cholecystokinin (CCK) became known not only in the gastrointestinal system but also in other organs as a hormone in metabolism control, a cytokine, and a neurotransmitter (Martin et al. 2020). Free fatty acids stimulate cholecystokinin release from the gut through GPR120-coupled Ca^2+^ signaling, and many other nutrients, amino acids, and bitter compounds increase CCK plasma levels (Tanaka et al. 2008). Many biologically active cholecystokinin proteins are secreted not only by intestinal cells but also in specific regions of the brain as neurotransmitters and in several endocrine glands, as well as the hypophysis, thyroid, pancreas islet, testis, and adrenal gland (Rehfeld 2017). Cholecystokinin proteins CCK-58, CCK-33, CCK-22, and CCK-8 are circulating in the blood, whereas the small CCK-8 and CCK-5 are potent neurotransmitters and bind to CCK-2 receptors in brain neurons (Rehfeld 2021). On this basis, it is not surprising that a growing number of publications describe CCK receptors in more and more tissues, and CCK receptors seem to be distributed widely in many organs and play a number of roles centrally and peripherally in the signaling network.

Two pharmacologically and molecularly different CCK receptor subtypes were identified and characterized. CCK-1R (named also CCK-A) needs the sulphated tyrosine moiety of CCK-8 and CCK-33 for binding. Both CCK-1R and CCK-2R consist of seven transmembrane protein domains and are members of the G-protein coupled superfamily of receptor molecules. CCK-2R (named also CCK-B) is less specific and binds both sulphated and non-sulphated cholecystokinin, gastrin, and CCK fragments (Alexander et al. 2011).

Cholecystokinin receptors seem to be involved in controlling of proliferation of many cells (Zeng et al. 2020). Otsuka Long-Evans Tokushima Fatty (OLETF) rats, originated from Long Evans Tokushima Otsuka (LETO) rats, have congenital defect by a 64-kb deletion in CCK-1R and a good animal model to study role of CCK-1R in various tissues. Hyperphagia and obesity with early onset are the most characteristic features of OLETF rats. After 18 weeks of age, hyperglycemia develops and about 40 weeks of age fibrosis in pancreas islets (Kawano et al. 1992; Miyasaka et al. 1994). Cholecystokinin protects beta cells of pancreas against apoptosis and can act as physiological growth factor in most parts of gastrointestinal tract (Lavine et al. 2015). When diabetes was induced by streptozotocin in CCK-1R and CCK-2R double-knock-out mice (CCK-1R^−/−^, −2R^−/−^) and in wide strain (CCK-1R^+/+^,−2R^+/+^), diabetic nephropathy was more serious in CCK double-knock-out than in the wide mice with more increased albuminuria, podocyte loss, and upregulated proinflammatory genes in the kidney. All findings suggested that diabetic renal injuries were exacerbated by the deletion of both CCK-1R and CCK-2R via the inflammatory process and CCK might have protective effect in kidney, too. Cholecystokinin is distributed in distal tubules and glomeruli in normal adult Sprague–Dawley rats, which also supports that CCK may have a role in kidney (Miyamoto et al. 2012).

Cholecystokinin receptors are important driving promoters of carcinogenesis in pancreatic cancer, and exogenous administration of CCK at physiological doses stimulates growth of human pancreatic cancer cells *in vitro* as well as in transplanted tumors *in vivo* in nude mice (Smith and Solomon 2014). Cholecystokinin has a role as stimulatory growth factor in numerous tumors, such as colon, gastric, pancreas, gallbladder, hepatocarcinoma, medullary thyroid carcinomas, small cell lung cancers, astrocytomas, meningiomas, and ovarian cancers (Reubi et al. 1997).

Cholecystokinin receptor antagonists have antiproliferative effects on many malignant cell lines and decrease growth of tumors *in vivo* in animal models and some clinical studies. Although proglumide is a weak antagonist with wide spectrum on CCK-1R, CCK-2R, and heterodimeric receptors, proglumide was able significantly to decrease growth of pancreatic intraepithelial neoplasm and the fibrosis associated with pancreatic cancer (Smith et al. 2014). In mice bearing subcutaneous murine hepatocarcinoma tumor cells, proglumide administration slowed the growth rate of these tumors (Gay et al. 2021). Immune checkpoint antibodies combined with proglumide, enhanced survival of human hepatocarcinoma bearing mice more, than without proglumide (Shivapurkar et al. 2023). Not only the non-selective CCK antagonists, but CCK-1R selective drugs were also successful, e.g., lorglumide in inhibition of carcinogenesis in pancreas (Watanapa et al. 1993) or devazepide against pancreas carcinoma in obese animal model (Matters et al. 2014). Some CCK-2R antagonists studied in clinical trials in pancreas cancer (Abbruzzese et al. 1992; Chau et al. 2006). Proglumide adjuvant therapy was used for patients with cardiac adenocarcinoma after gastrectomy for 5 years, and the 1-, 5-, 10-year survival rates were better than in patients with surgery without proglumide treatment (Chen et al. 2005). Other tumors bearing CCK receptors maybe also targets for CCK antagonists.

Cholecystokinin antagonists are investigated for use in protocols against some malignancies. During cancer treatment protocols, myelotoxicity often results in dose modifications, delays, or discontinuation of therapy. The effect of cholecystokinin antagonists on hemopoiesis has not been studied; however, the immunomodulatory effects of cholecystokinin became increasingly obvious and are associated with CCK-1 receptors (Batty et al. 2021). Mature neutrophils and macrophages, which originate from granulocyte–macrophage progenitor cells (CFU-GM) of bone marrow during granulopoiesis, could respond to cholecystokinin and CCK antagonists (De la Fuente et al. 2000; Saia et al. 2014). The role of cholecystokinin in hemopoiesis, including granulopoiesis, is less known; however, CCK-2R is found in myeloid and lymphoid leukemic cell lines and participates in autocrine loops (Iwata et al. 1996). Antagonists of CCK-2R were tested as antileukemic agents (Stubbs et al. 2005). Miyamoto et al. (2012) found that damage to the diabetic kidney involving inflammation caused by bone marrow-derived macrophages was more serious if these wild mice (CCK-1R^+/+^, −2R^+/+^) underwent bone marrow transplantation with cells from animals with CCK-1 receptor deficiency (CCK-1R^−/−^ mice). These results suggested that cholecystokinin may have some role in granulopoiesis via CCK-1R.

We would like to study hemopoiesis of CCK-1R deficient OLETF and their counterpart wild LETO rats, as there are data about CCK receptors in macrophages, but there are no data about CCK receptors in granulocyte–macrophage progenitors (CFU-GM) of normal bone marrow. It has importance when CCK antagonists are tried to be used in cancer treatment protocols. A question arises whether CCK antagonists influence CFU-GM in granulopoiesis, which is one of the key targets of myelotoxicity of anticancer drugs. Effects of both CCK non-selective and CCK-1R selective antagonists, namely proglumide and lorglumide, were studied. Carboplatin is widely used in solid tumors, including pancreas and hepatocarcinomas (Azmy et al. 2012; Chaigneau et al. 2023). To study whether these CCK antagonists may influence chemotherapy, we tested their effects on the toxicity of carboplatin.

## Materials and methods

### Animals and ethical approval

Long Evans Tokushima Otsuka (LETO) and Otsuka Long Evans Tokushima Fatty (OLETF) rats were kindly provided by Kazuya Kawano from Tokushima Research Institute (Otsuka Pharmaceutical, Tokushima, Japan). All rats were males and 8 weeks old. The body weights of LETO rats were 256 ± 28 g, and the body weights of OLETF rats were significantly higher, 331 ± 34 g (*P* < 0.01). Blood sugar levels were in the normal range in both groups. The rats were fasted overnight with allowed access to tap water “ad libitum” before blood glucose concentration was determined by glucometer (Accu-Chek, Roche Diagnostics, Budaörs, Hungary).

The animals were housed in an animal room with 12-h light and dark periods a day, a temperature of 23 ± 2 °C, a relative humidity of 60 ± 10% with 3–5 animals per pen. They were fed commercial laboratory chow and tap water ad libitum. Rats were treated ethically and sparingly, and all methods in this study were conducted according to the “Principles of Laboratory Animal Care” outlined in EU Directive 2010/63/EU. The local Ethics Committee of the University of Debrecen approved all experimental protocols (approval number: 13/2022/DEMÁB).

### Study design

Hemopoiesis was studied in the femoral bone marrow of 8-week-old obese OLETF and wild nonobese LETO rats. Altogether 10 OLETF and 10 LETO rats were used. After extermination of animals by an intravenous overdose with 100 mg/kg of thiopental sodium (Thiopental Sandoz, Sandoz Pharmaceutical PLC, Switzerland), femoral bones of the rats were prepared under sterile conditions, and the bone marrow was completely washed out by McCoy’s 5 A medium (Sigma-Aldrich Merck KGaA, Darmstadt, Germany). Single cell suspensions, checked under microscope, were obtained by suspending bone marrow samples in McCoy’s 5 A medium through thin needle syringes. After Ficoll-Iodamide gradient centrifugation, the mononuclear cell fraction of bone marrow was used (see under CFU-GM assay).

Bone marrow function was characterized by measuring total femoral cellularity, colony numbers of CFU-GM, and total femoral CFU-GM content. Cellularity of the whole femoral bone marrow shows the number of the total mononuclear bone marrow cells, which is a good indicator of the intensity of hemopoiesis, including all cell lineages. Cellularity was calculated using bone marrow cell counts and volumes of samples. Colony numbers of CFU-GM progenitors were counted in special soft-gel cultures prepared according to the CFU-GM colony assay, and the total CFU-GM content of the femur was calculated by multiplying cellularity and frequency of CFU-GM indicated by colony numbers. The latter values mirror the intensity of granulopoiesis.

The bone marrow mononuclear cells of each rat were grown in normal conditions or in the presence of carboplatin at final concentrations in 0.2–3 mg/L range. Carboplatin (Sigma-Aldrich Merck KGaA, Darmstadt, Germany) was dissolved in sterile distilled water at 50 °C in an ultrasonic bath (XUB5, Bio-Science Ltd., Budapest, Hungary) freshly in each experiment. When it completely dissolved, this solution of 1 mg/ml concentration was immediately diluted 100 times by culture medium and used for the CFU-GM assay.

In the further experiments Long Evans Tokushima Otsuka (LETO) rats were treated with CCK antagonists using equimolar doses at two levels of both proglumide and lorglumide. After 1-week acclimatization period, the rats were randomly allocated into five groups (*n* = 12/group): first group receiving vehicle (0.9% NaCl) treatment, the next two groups receiving 3 mg/kg or 10 mg/kg of proglumide (proglumide sodium, Mw: 356.39 g/mol, Sigma-Aldrich Merck KGaA, Darmstadt, Germany), and the other two groups receiving 4 mg/kg or 13 mg/kg of lorglumide (lorglumide sodium, Mw: 481.39 g/mol, Sigma-Aldrich Merck KGaA, Darmstadt, Germany) once daily intravenously for 5 consecutive days. The used doses were $8.4\cdot {10}^{-6}$ mol/kg and $2.7\cdot {10}^{-5}$ mol/kg equimolar doses of proglumide and lorglumide with the same number of molecules at the dose levels, in the interest of the accurate comparison of their effects. Pharmacological effects can be compared at molecular levels on receptors if the same number of molecules of the studied drugs are used. Cholecystokinin antagonists were administered intravenously as a slow bolus injection in 3–4 s using special one-rat cage after submersing the tail in warm water for 1–2 min. We administered the proper doses each day at the same time with the exact same order for the rats starting at 9.00.

On the 6th day, the animals were exterminated by an intravenous overdose 100 mg/kg of thiopental sodium (Thiopental Sandoz, Sandoz Pharmaceutical PLC, Switzerland). Bone marrow was obtained and handled similarly than in the previous study to see effects of CCK antagonists on hemo- and granulopoiesis. To study whether the pre-treatment with the cholecystokinin antagonists influences sensitivity of CFU-GM progenitors against carboplatin toxicity, the bone marrow mononuclear cells of each rat were grown in normal conditions or in the presence of carboplatin of 1 or 2 mg/L final concentrations. Carboplatin was dissolved freshly in each experiment and handled similarly as previously described.

### CFU-GM assay

The soft-gel cultures were prepared to maintain descendants of CFU-GM progenitor cells together, which formed colonies and showed the frequency of CFU-GM among the inoculated bone marrow cells as described previously (Benkő et al. 1999). Briefly, bone marrow cell suspensions diluted 0.9% NaCl in a 1:1 ratio, and mononuclear cell fractions were obtained by Ficoll-Iodamide (Sigma-Aldrich Merck KGaA, Darmstadt, Germany) gradient centrifugation (1077 g/mL). Viable cells of the buffy coat were counted with the help of a hemocytometer, using 0.04% trypan blue; viability was consistently 99–100%. Inocula of 1 × 10^5^/mL rat bone marrow cells were plated in petri dishes with a diameter of 35 mm (Greiner, Nürtingen, Germany) and were grown in McCoy’s 5 A modified medium (Sigma-Aldrich Merck KGaA, Darmstadt, Germany) supplemented with amino acids, Na pyruvate, NaHCO_3_, antibiotics (streptomycin, penicillin), 20% fetal bovine serum (Sigma-Aldrich Merck KGaA, Darmstadt, Germany), and colony-stimulating factors, namely rrGM-CSF and rhG-CSF (Sigma-Aldrich Merck KGaA, Darmstadt, Germany). Methylcellulose (Methocel, 3000–5000 centipoises; FLUKA, Buchs, Switzerland) at 1.2% was used as a support matrix for semisolid cultures. The cultures were grown in duplicates for 14 days at 37 °C at 100% relative humidity in an atmosphere containing 5% CO_2_. The colonies were counted under a dissecting microscope (Olympus, Hamburg, Germany) at the end of the incubation period. Colonies were defined as groups of at least 50 cells, consisting of granulocytes and/or monocytes, verified by smears or cytospin preparations.

### *In vitro* treatment of CFU-GM progenitors of LETO rats

To see whether the effects of CCK antagonists were direct or indirect on the progenitor cells, samples of a further 10 LETO rats were used. Primary cell cultures were made from sterile bone marrow mononuclear cells of non-obese LETO rats according to the previously described method, and in these soft gel cultures, CFU-GM progenitors were grown without or with proglumide or lorglumide using 12 concentration levels in a 0.1 to 15,000 $\mu$M range.

### Testing CCK-1 and CCK-2 receptors in bone marrow cells

Receptors of CCK-1 and CCK-2 were tested in femoral mononuclear bone marrow cells of LETO and OLETF rats (*n* = 10/group) treated with vehicle (0.9% NaCl) or 10 mg/kg of proglumide once daily orally for 4 weeks. We mixed samples of 2 rats to get enough amounts of cells to study; thus, data are originated from 5–5 samples. Then, the mononuclear bone marrow cells of the two groups of rats were cultured in modified McCoy’s 5 A medium according to the previously described CFU-GM assay with colony-stimulating factors but without methylcellulose in suspension culture flasks (Greiner Bio-One Ltd. Hungary, Mosonmagyaróvár, Hungary). From the single-cell bone marrow, suspensions mononucleated cells were obtained from buffy coat after Ficoll-Iodamide gradient centrifugation (1077 g/mL) and counted by hemocytometer using trypan blue. Viable nucleated cells of 5 × 10^6^/mL were seeded into culture flasks (25 mL) containing 10 mL of abovementioned completed medium and were grown at 37 °C in an atmosphere containing 5% CO_2_ with 100% relative humidity.

The progenitor cells have the highest proliferation rate among mononuclear bone marrow cells, and after a few days, their numbers exponentially increase. The special colony-stimulating factors of the CFU-GM assay are required for CFU-GM progenitors to survive and differentiate, and at the same time, the other cells have been much less supported. Missing methylcellulose resulted in less stimulation for CFU-GM to differentiate. Cytospin preparations of the cells in liquid cultures were checked day by day, and on the fifth day, we found increased cell numbers, which showed mononucleated cells, including some myeloblasts, without any more differentiated cells or cells with segmented nuclei. We used the non-adherent cells from these liquid cultures on the 5th day, carefully paying attention to exclude adherent stromal or more differentiated other cells. As a result of this method, we got CFU-GM enriched bone marrow cell suspension.

### RNA extraction and reverse-transcription

Bone marrow cells derived from abovementioned cultures were hold in RNA later solution (Qiagen, Hilden, Germany) which prevents degradation of mRNA until RNA extraction. Total RNA was purified using RNA Protect Mini kit (Qiagen) according to the manufacturer’s recommendations. Concentration of RNA was determined by SmartSpec™ Plus Spectrophotometer (Bio-Rad, Hercules, USA). First-strand cDNA synthesis was performed on 2 μg of total RNA using random primers (Invitrogen Life Technologies, Karlsruhe, Germany) and Superscript™ II RNase H- Reverse Transcriptase (Invitrogen) as described previously (Varga et al. 2007).

### Real-time quantitative PCR

Real-time PCR was performed using ABI PRISM® 7900 HT Sequence Detection System (Applied Biosystems Inc, Foster City, CA, USA) in 10-μl aliquots containing 2 × FastStart® TaqMan Master Mix (Rox) (Rosche Applied Science, Mannheim, Germany), 4.5 μl cDNA template and either 0.5 μl TaqMan® Gene Expression inventoried assay (for CCK-1 and CCK-2 receptor) or oligos for 18S rRNA (500 nM forward and reverse primer each plus 100 nM probe). The assay probes contain a 6-carboxy-fluorescein phosphoramidite (FAM dye) labeling at the 5′ end of the probe and a minor groove binder plus a non-fluorescent quencher at the 3′ end, while the probe for 18S rRNA was labeled with TAMRA as a quencher instead of MGB. TaqMan® Gene Expression Assay IDs were Rn00562164_m1 for CCK-1R (Acc. No.: NM_012688) and Rn00565867_m1 for CCK-2R (Acc. No.: NM_013165), respectively. The expression of the housekeeping gene 18S rRNA (Acc. No.: EF060919) served as internal control. The oligonucleotide sequences for 18S rRNA were designed using qPCR primer version 1.2 and ordered from Bio-Science Ltd (Budapest, Hungary). Amplification conditions consisted of 95 °C for 10 min, followed by 40 cycles of 95 °C for 15 s (denaturation) and 60 °C for 1 min (annealing and amplification) in an ABI PRISM® 7900 HT Sequence Detection System (Applied Biosystems).

### TaqMan assay assessment

TaqMan PCR for target (CCK-1R and CCK-2R) and reference (18S rRNA) genes was run from the same RT reaction. Three replicates were run for each gene for each sample in a 384-well format plate. “No template controls” (NTC) were used to confirm that reagents were not contaminated. C_t_ values were calculated with the Applied Biosystems SDS 2.1 software and were analyzed using the threshold cycle (*C*_t_) relative quantification method (Livak and Schmittgen 2001).

### Statistical analysis

The data obtained from individual rats or samples were used for statistical analysis. The results are presented as means ± standard error of the mean (S.E.M). Differences among the groups were analyzed using Kruskal–Wallis analysis of variance followed by the Mann–Whitney *U* test for comparison of two groups or samples. Differences were regarded as statistically significant at *P* < 0.05 and signed by stars (*P < 0.05, **P < 0.01, *** P < 0.001, ****P < 0.0001). We used the statistical package of GraphPad Prism software for Windows (version 8.0.1 GraphPad Software Inc., La Jolla, CA, USA).

For mRNA expression, data were analyzed by comparing the means of the 2^(−ΔΔCT)^ values. The levels of CCK-1 and CCK-2 receptor transcripts were normalized to the levels of 18S rRNA (ΔCT). Then, ΔCT was normalized to the values of untreated LETO and OLETF rats (ΔΔCT). The normalized ΔCT values for untreated LETO or OLETF rats were considered as 1 (100%). All 2^(−ΔΔCT)^ values were expressed as the mean ± S.E.M. and were evaluated statistically by Kruskal–Wallis analysis of variance followed by the Mann–Whitney *U* test. Differences were regarded as statistically significant at *P* < 0.05 from its corresponding value of untreated control rats at both genes examined.

## Results

### Hemopoiesis in obese OLETF rats with CCK-1R deficiency and non-obese LETO rats

**Fig. 1:**
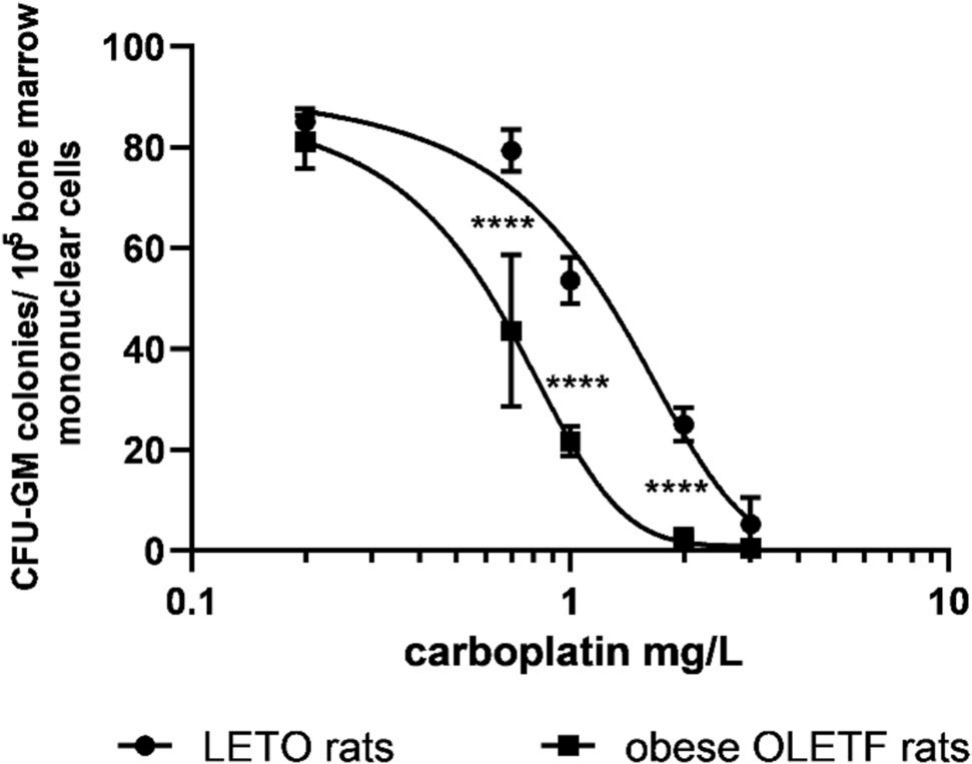
Sensitivity of CFU-GM progenitors to carboplatin in obese OLETF and non-obese LETO rats. Colony numbers of CFU-GM were determined when colonies were grown in the presence of carboplatin. Data are expressed as mean ± S.E.M. (****P < 0.0001 colony numbers of obese OLETF rats compared to the corresponding data of the control non-obese LETO rats; *n* = 10)

We studied OLETF rats to see whether the CCK-1R deficiency influences hemopoiesis. Bone marrow function was evaluated by total cellularity, CFU-GM colony formation, and CFU-GM content of femoral bone marrow. Neither cellularity nor CFU-GM colony numbers or the total femoral CFU-GM content of OLETF rats did not significantly differ from those of wild LETO rats from which OLETF rats originated. However, when we tested toxicity of carboplatin *in vitro*, decreased threshold was evaluated in CFU-GM. Dose–response curve of carboplatin was shifted to the left, and IC_50_ of carboplatin was 0.62 mg/L in CFU-GM of obese OLETF vs. 1.16 mg/L in CFU-GM of non-obese LETO rats (Fig. 1).

### Effect of proglumide and lorglumide on CFU-GM progenitors of bone marrow *in vivo*

**Fig. 2:**
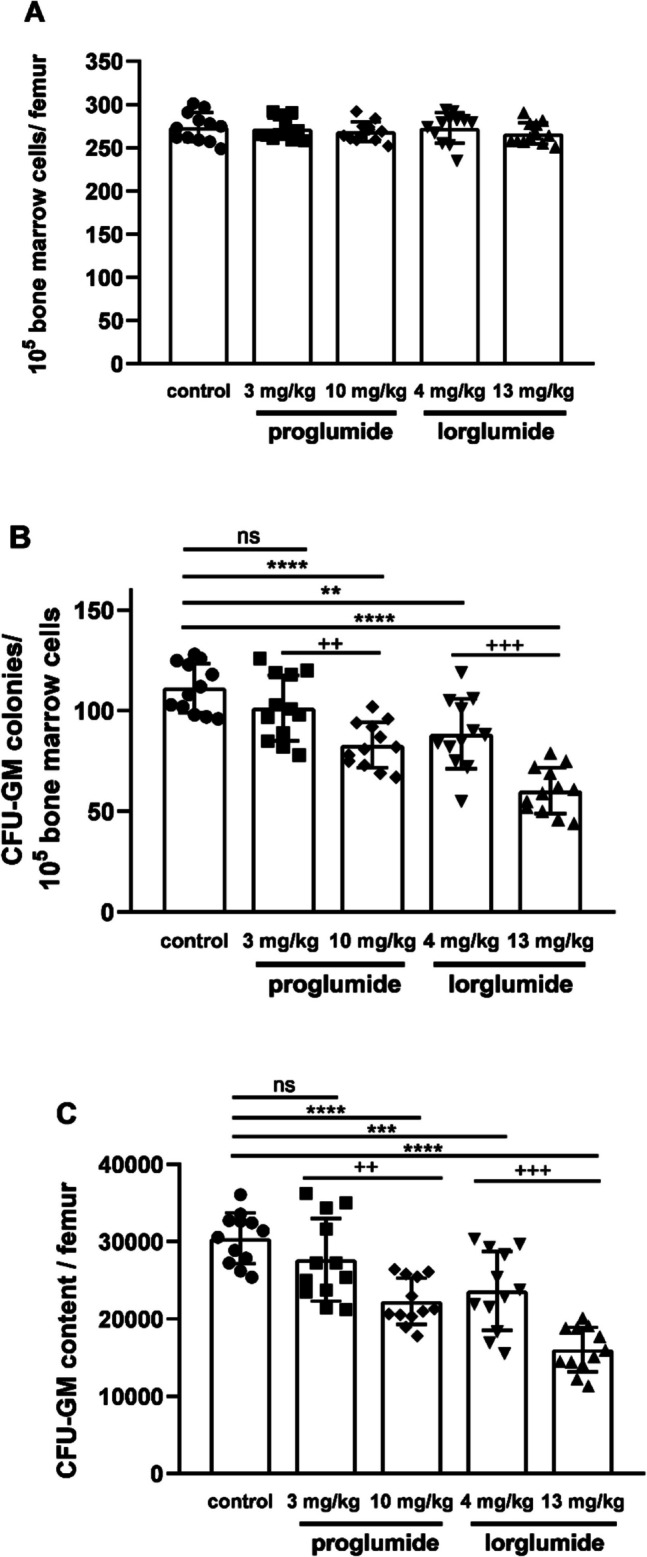
Effect of proglumide and lorglumide treatment *in vivo* on hemopoiesis characterized by cellularity (**A**), CFU-GM colony numbers (**B**), and content of femoral bone marrow (**C**) in LETO rats. For rats in control group vehicle and in the other groups equimolar doses of CCK antagonists, 3 mg/kg or 10 mg/kg of proglumide and 4 mg/kg or 13 mg/kg lorglumide were administered intravenously for 5 consecutive days. **P < 0.01; ***P < 0.001; ****P < 0.0001 compared to vehicle-treated control LETO rats; + + *P* < 0.01, + + + *P* < 0.001 differences between the two studied doses of each drug; *n* = 12/group. Data are expressed as mean ± S.E.M

Cellularity of femoral bone marrow shows total numbers of mononuclear cells in femoral bone marrow of rats including all type of hemopoietic and stromal stem cells with their descendants in hemopoietic lineages. Both CCK antagonists did not changed cellularities of LETO rats (Fig. 2A). Nevertheless both CCK antagonists damaged granulopoiesis *in vivo.* Differentiating granulocytes are not included in mononuclear cells of bone marrow; however, their progenitors, the granulocyte–macrophage colony forming unit (CFU-GM), is included, as one of the populations of stem cells. Frequencies of CFU-GM as well as their total numbers in femur were less, than in control animals at $2.7\cdot {10}^{-5}$ mol/kg, the higher equimolar doses of proglumide (10 mg/kg, *P* < 0.0001) and lorglumide (13 mg/kg, *P* < 0.0001). In addition, CCK-1R-selective lorglumide decreased CFU-GM population also in the lower dose in 4 mg/kg, while proglumide had no effect in the equimolar 3 mg/kg dose (Fig. 2B, C).

### Effect of proglumide and lorglumide pre-treatment *in vivo* on *in vitro* toxicity of carboplatin to CFU-GM progenitors

**Fig. 3:**
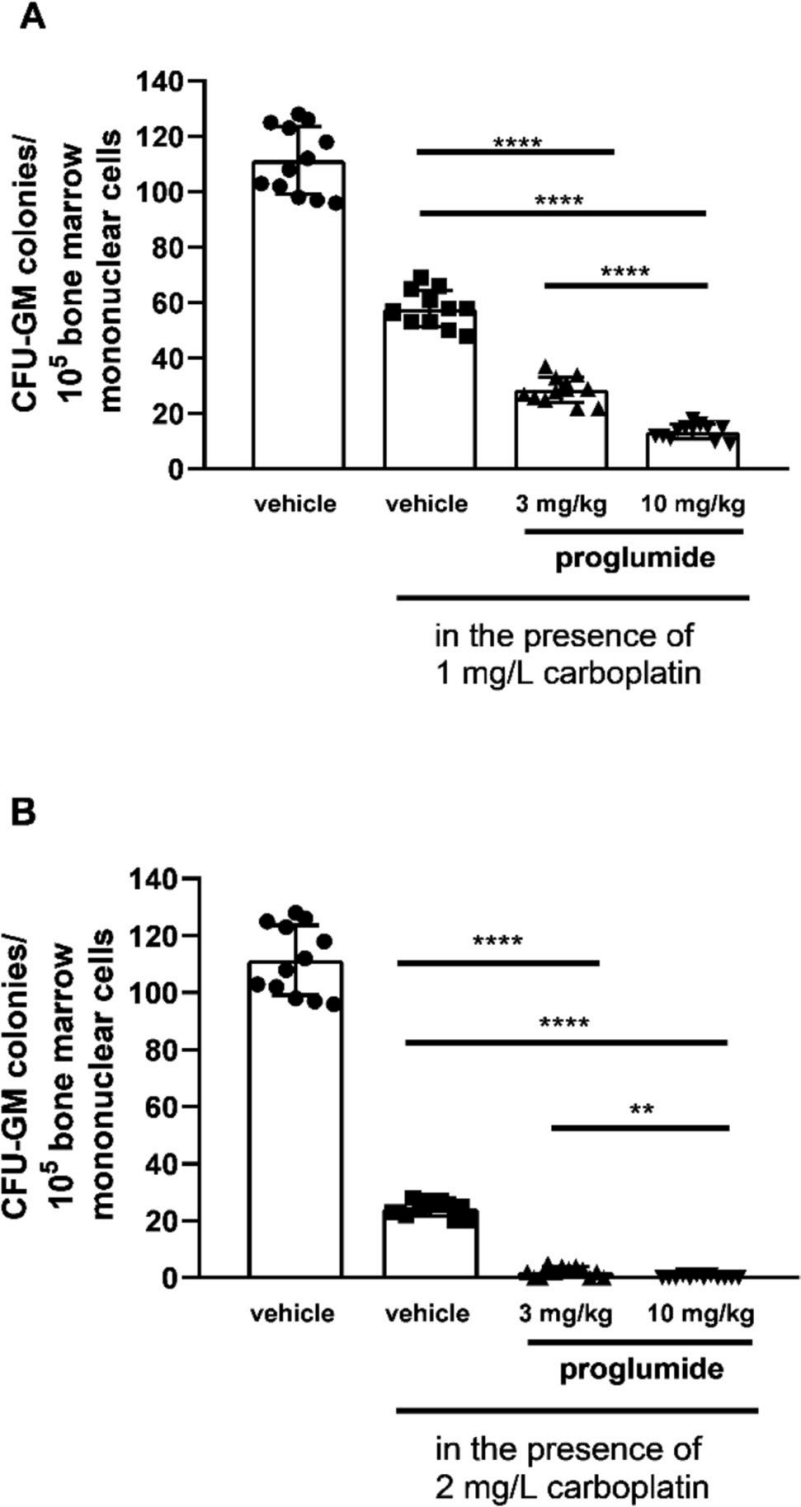
Sensitivity of CFU-GM progenitors to carboplatin in wild non-obese LETO rats pre-treated with proglumide, a non-selective CCK antagonist *in vivo*. First columns show CFU-GM colony numbers of control rats treated with vehicle *in vivo* and CFU-GM colony numbers were determined when colonies were grown without carboplatin. Second columns show colony numbers of cultures, in which bone marrow mononuclear cells originated from control rats were grown in the presence of carboplatin in 1 mg/L concentration (**A**) and in 2 mg/L concentration (**B**). Third and fourth columns show CFU-GM colony numbers determined of cultures, in which cells originated from rats pretreated with proglumide, were cultured in the presence of carboplatin in 1 mg/L (**A**) or 2 mg/L (**B**) concentrations. Data are expressed as mean ± S.E.M. (*n* = 12; ****P < 0.0001 compared to colony numbers of cultures from vehicle-treated LETO rats, in which the bone marrow cells were grown in the presence of carboplatin (second columns)

**Fig. 4:**
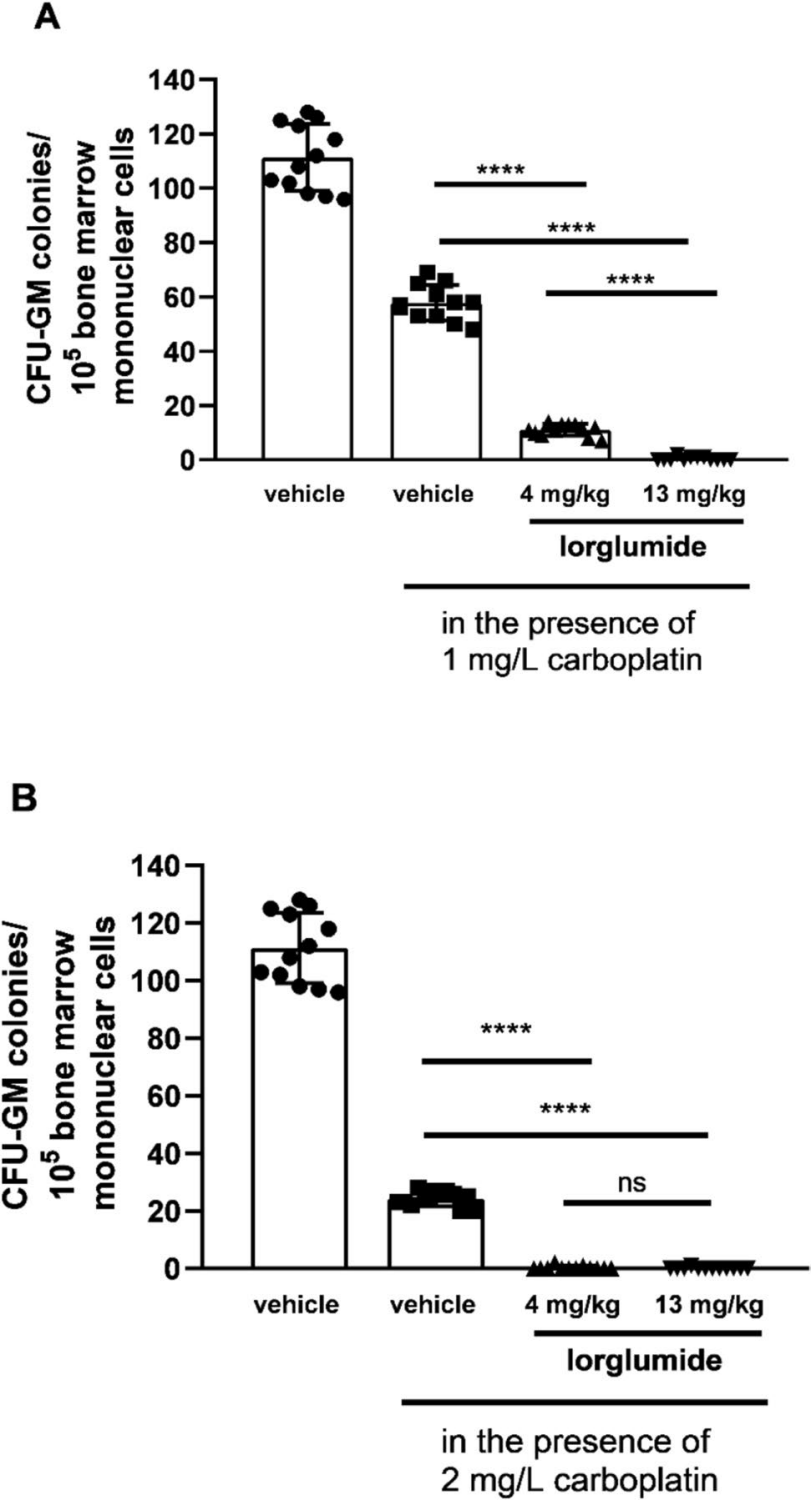
Sensitivity of CFU-GM progenitors to carboplatin in wild non-obese LETO rats pre-treated with lorglumide, a CCK-1 receptor selective CCK antagonist *in vivo*. First columns show CFU-GM colony numbers of control rats treated with vehicle *in vivo*, and CFU-GM colony numbers were determined when colonies were grown without carboplatin. Second columns show colony numbers of cultures in which bone marrow mononuclear cells originated from control rats were grown in the presence of carboplatin in 1 mg/L concentration (**A**) and in 2 mg/L concentration (**B**). Third and fourth columns show CFU-GM colony numbers determined of cultures in which cells originated from rats pretreated with lorglumide were cultured in the presence of carboplatin in 1 mg/L (**A**) or 2 mg/L (**B**) concentrations. Data are expressed as mean ± S.E.M. (*n* = 12; ****P < 0.0001 compared to colony numbers of cultures from vehicle-treated LETO rats, in which the bone marrow cells were grown in the presence of carboplatin (second columns)

Carboplatin decreased CFU-GM colony numbers dose-dependently in bone marrow cultures of LETO rats. The second columns of Figs. 3 and 4 show colony numbers of cultures, in which bone marrow mononuclear cells were cultured in the presence of carboplatin in 1 mg/L concentration (Figs. 3A, 4A) and 2 mg/L concentration (Figs. 3B, 4B) when cells were obtained from control LETO rats treated with vehicle *in vivo* (Fig. 3A and B, *P* < 0.0001, and Fig. 4A and B, *P* < *0.0001*). We found increased sensitivity of CFU-GM progenitors against carboplatin after 5-day-long administration both with proglumide (Fig. 3) and lorglumide (Fig. 4) used in equimolar doses (proglumide, 10 mg/kg; and lorglumide, 13 mg/kg, respectively). After both proglumide and lorglumide treatment of LETO rats, we observed less CFU-GM colony numbers dose-dependently, comparing with the vehicle-treated control LETO rats using *in vitro* colony assay, in which CFU-GM cells were grown in the presence of carboplatin with 1 mg/L (Fig. 3A and Fig. 4A, *P* < 0.0001 for each corresponding pair) or 2 mg/L (Fig. 3B and Fig. 4B, *P* < 0.0001) concentrations. Carboplatin in 1 mg/L concentration decreased colony numbers of CFU-GM to 28% in cultures of rats pre-treated with proglumide 3 mg/kg comparing with 11% in cultures of rats pre-treated with lorglumide equivalent 4 mg/kg dose (Fig. 3A and Fig. 4A, *P* < *0.0001*). At the same time, carboplatin in 1 mg/L concentration inhibited proliferation of CFU-GM progenitors of the animals pre-treated with lorglumide completely, while 13% of CFU-GM survived after proglumide pre-treatment using $2.7\cdot {10}^{-5}$ mol/kg, the higher equimolar doses of proglumide (10 mg/kg, *P* < 0.0001) and lorglumide (13 mg/kg, *P* < 0.0001) (Fig. 3A and Fig. 4A). The CCK-1R selective lorglumide had more powerful sensitizing effect to carboplatin toxicity than the non-selective proglumide.

### Direct effect of proglumide and lorglumide on CFU-GM progenitors of LETO rats *in vitro*

**Fig. 5:**
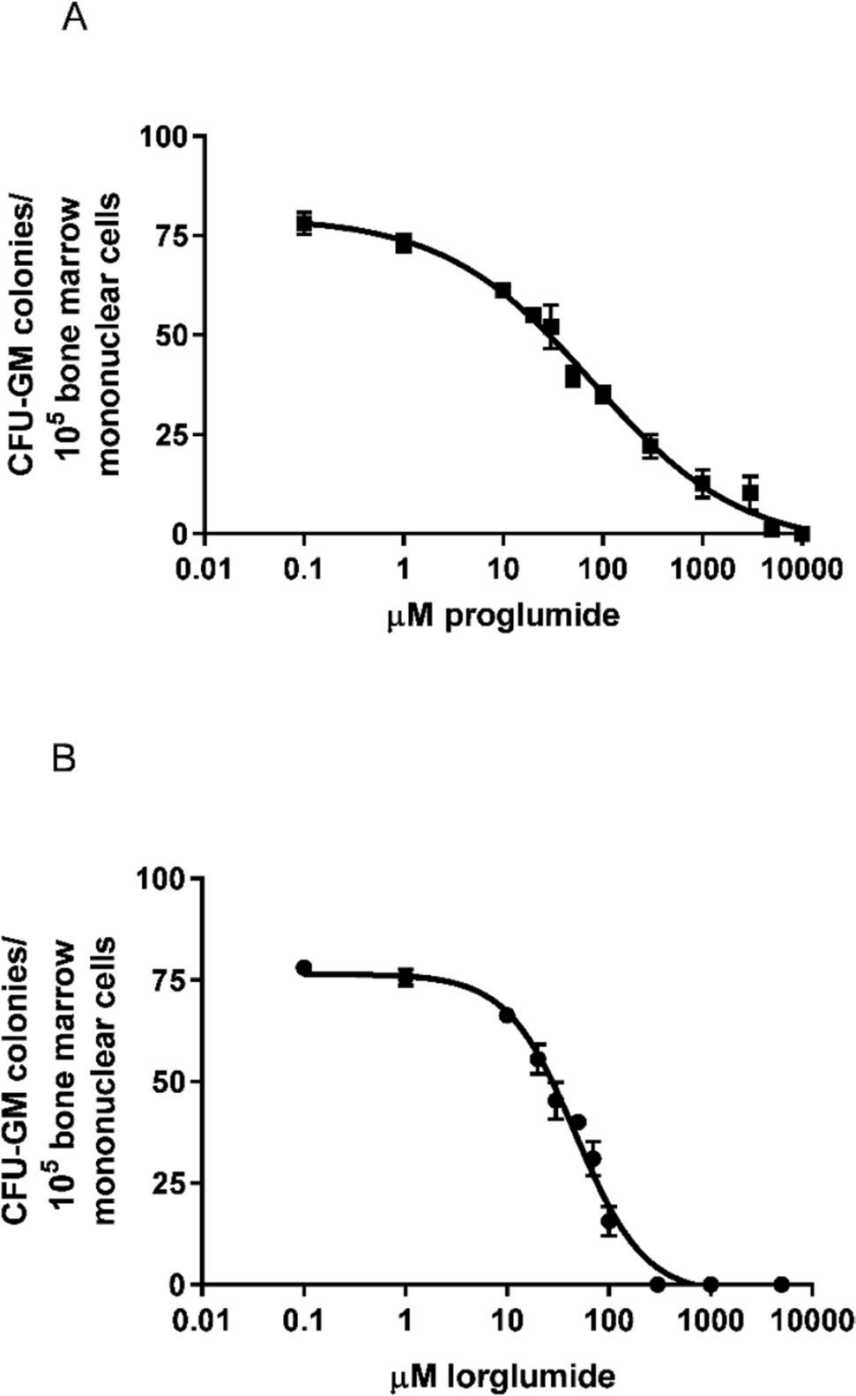
Effect of proglumide and lorglumide on CFU-GM progenitors of wild non-obese LETO rats *in vitro*. Proliferation of CFU-GM progenitors was dose dependently inhibited by culturing them in the presence of CCK antagonists. The IC50 values were 76.82 $\mu$M for proglumide and 47.37 $\mu$M for lorglumide, which are non-selective and CCK-1R selective antagonists, respectively. Colony numbers of CFU-GM were determined when colonies were grown without CCK antagonists (control) or in the presence of proglumide (**A**) or lorglumide (**B**). Data are expressed as mean ± S.E.M. (*n* = 10)

If we cultured bone marrow mononuclear cells of LETO rats in the presence of proglumide and lorglumide in the proper dose ranges, inhibitory effects were evaluated dose-dependently on the proliferation of CFU-GM cells of the LETO rats. It is a result of direct effects on the progenitor cell population. The CCK-1R-selective lorglumide caused more powerful inhibition, than the non-selective proglumide. Dose–response curve of lorglumide was steeper and half maximal inhibitory concentrations, the IC50 values were 76.82 and 47.37 $\mu$M for proglumide and lorglumide, respectively (Fig. 5).

### Testing cholecystokinin receptors in bone marrow cells

For quantification of messenger RNA (mRNA) transcripts, RT-PCR method was used, and we found that both CCK-1 and CCK-2 receptors were expressed in the progenitor cells of bone marrow of LETO rats. We also used OLETF rats with CCK-1R deficiency and CCK-2 receptors were found without CCK-1 receptors on their progenitor cells.

**Fig. 6:**
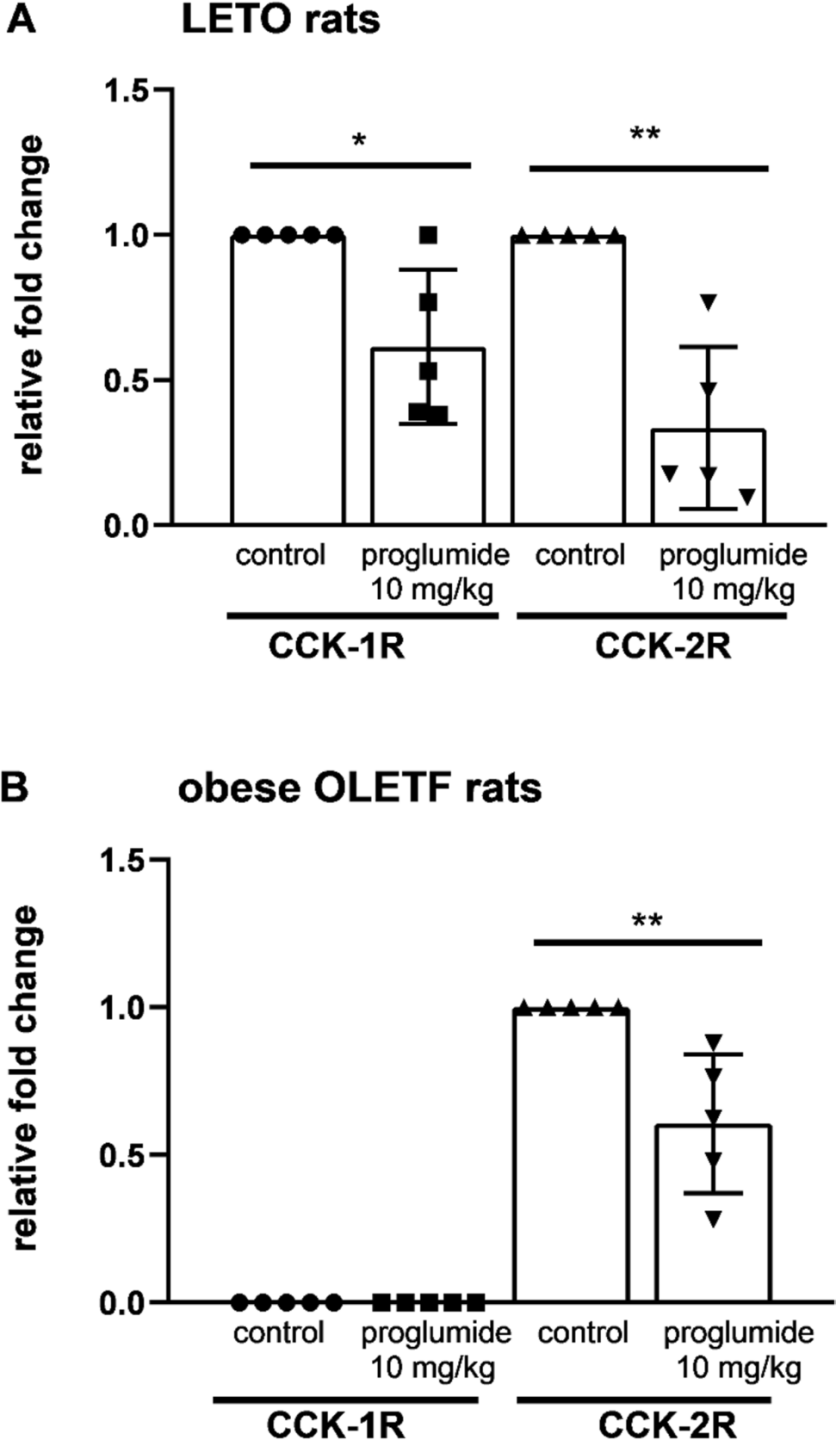
Both CCK-1 and CCK-2 receptors were found in bone marrow progenitor cells of wild non-obese LETO rats (**A**). CCK-2R was found in obese OLETF rats with CCK-1R deficiency (**B**). Relative mRNA levels of both CCK-1R and CCK-2R were significantly downregulated after proglumide treatment in a 10 mg/kg daily dose orally for 4 weeks compared to data from samples of non-obese LETO controls treated with vehicle. In obese OLETF CCK-1R-deficient rats, CCK-1R was not detected. CCK-2R was also downregulated by proglumide in OLETF rats treated in the same way. (*P < 0.05, **P < 0.01 between samples of untreated and proglumide-treated groups; *n* = 5). Data are expressed as mean ± S.E.M

Treatment with proglumide, a non-specific CCK antagonist in 10 mg/kg daily doses administered for 4 weeks, significantly downregulated relative mRNA levels of both CCK-1R and CCK-2R in bone marrow progenitors of the proglumide-treated LETO rats in comparison with their untreated controls (Fig. 6A). We were able to detect only CCK-2R in bone marrow progenitor cells of OLETF rats treated with proglumide by the same manner, and decrease in CCK-2 receptors was also significant after proglumide administration in OLETF rats (Fig. 6B).

## Discussion

Myelotoxicity is a dose-limiting side effect of chemotherapy that contributes to the narrow therapeutic windows of anticancer drugs. Each hemopoietic cell lineage is affected; however, the granulocyte–macrophage progenitor population (CFU-GM) is one of the key targets, which limits the use of the proper doses of treatment protocols because of the risk of serious neutropenia. Cells of this population are the common progenitors of granulocytes and monocytes, including phagocytes and macrophages for non-specific immune responses. The progenitor cell population has a higher proliferation rate than that of stem cell populations, which results in their increased damage during antiproliferative therapy by anticancer drugs. Myelosuppression with prolonged and more serious neutropenia associated with infections is more frequent during the chemotherapy of obese patients than in those who have normal weights (Petrelli et al. 2021). Cholecystokinin antagonists are investigated for use against the pancreas and hepatocarcinomas, the risks of which are higher with other malignancies in obesity, with poorer prognosis than in nonobese patients (Gupta et al. 2018; Xu et al. 2018).

Insulin, leptin, and cholecystokinin are implicated in the control of satiety, and their dysfunctions are associated with the development of obesity and its complications (Chang 2022; Vilarino-Garcia et al. 2024). Many cytokines drive and support the proliferation, differentiation, and survival of CFU-GM progenitors and their precursor stem cells. The names of granulocyte–macrophage and granulocyte colony-stimulating factors (GM-CSF; G-CSF) are originated from their importance in granulopoiesis. It is less known that insulin and leptin also stimulate and protect hemopoietic stem cells, as well as the wide range of progenitors including CFU-GM (Claycombe et al. 2008; Machado-Neto et al. 2018). Leptin stimulates cholecystokinin release from the gut; cholecystokinin influences the distribution of leptin and regulates insulin release from pancreas, and among them, synergistic effects are common in many tissues (Guilmeau et al. 2003; Cano et al. 2003). We studied whether CCK also influences hemopoiesis.

Role of cholecystokinin has not known in hemopoiesis; however, Miyamoto et al. (2012) found that transplantation with bone marrow, derived from mouse with CCK-1 receptor deficiency, produced proinflammatory macrophages and aggravated damage in diabetic kidney, which suggested that cholecystokinin influences granulopoiesis via CCK-1R signaling. We studied hemopoiesis of Otsuka Long-Evans Tokushima Fatty (OLETF) rats with CCK-1 deficiency and LETO rats from which OLETF rats originated. OLETF rats have been established as an animal model of obesity and non-insulin dependent diabetes mellitus (Miyasaka et al. 1994). In our previous experiments, myelotoxicity of carboplatin was increased *in vivo* in db/db mice, the animal model of obesity-associated diabetes mellitus (Géresi et al. 2015). Diabetes mellitus develops in OLETF rats about 18 weeks of age; therefore, we used 8-week-old rats with normal fasting blood glucose values; however, insulin resistance cannot be completely excluded, as they already had overweight. Although hemopoiesis seemed to be intact in the obese OLETF rats on the first look, sensitivity of their CFU-GM progenitors was significantly greater to toxicity of carboplatin, than that of LETO rats (Fig. 1). Insulin resistance itself also decreases threshold of sensitivity against toxicity of carboplatin (Géresi et al. 2012), that is why in the further experiments we used wild non-obese LETO rats to see whether CCK antagonists have effect on granulopoiesis via CCK receptors.

In our previous experiments, proglumide inhibited postprandial whole body insulin sensitization above 10 mg/kg dose (Peitl et al. 2010), and lorglumide inhibited cerulein-induced amylase secretion of the pancreas in 10 mg/kg dose in rats (Scarpignato et al. 1989). That is why we used 10 mg/kg proglumide and 13 mg/kg lorglumide equimolar doses with the same number of molecules, which seemed to be a dose affecting physiological processes in rats. To differentiate indirect effects of insulin sensitization/resistance and direct effects of CCK antagonists on bone marrow cells, we used also a lower $8.4\cdot {10}^{-6}$ mol/kg dose, which is equal to 3 mg/kg of proglumide and 4 mg/kg of lorglumide, and furthermore, we studied direct effects of proglumide and lorglumide *in vitro* in bone marrow cell cultures, too. We found inhibitory effects of CCK antagonists on CFU-GM both *in vivo* (Fig. 2) and *in vitro* (Fig. 5). Not only the non-selective proglumide, but also the CCK-1R-selective lorglumide damaged CFU-GM cells; in addition, lorglumide had more powerful effect than proglumide. Lorglumide damaged granulopoiesis in 4 mg/kg dose, while proglumide had no effect in the equimolar dose. In higher doses, both CCK antagonists inhibited CFU-GM cells (Fig. 2). Cholecystokinin antagonists inhibited CFU-GM progenitor cells dose-dependent manner *in vitro*, too, which confirmed direct effects on CFU-GM progenitors; IC50s were 47.37 vs. 76.82 $\mu$M, respectively (Fig. 5).

However, both CCK receptors have a role in carcinogenesis in pancreas malignancies; the CCK-1 receptor might have a more specific role, as the highly selective CCK-2R antagonist, Z-360, had no effects in patients with metastatic pancreatic cancer in a phase II study (Ueno et al. 2017). Both proglumide and lorglumide were successful against pancreas carcinoma in animal experiments *in vivo* (Smith et al. 2014; Watanapa et al. 1993). Devazepide, a CCK-1R selective antagonist with a different chemical structure than lorglumide and proglumide, was also successful in decreasing the invasiveness of pancreatic cancer cells in mice (Matters et al. 2014), which suggested a specific effect via CCK-1R. All in all, the effective antitumoral doses from animal experiments corresponded to those that inhibited the proliferation of CFU-GM in normal bone marrow in our experiments. Lorglumide inhibited carcinogenesis at a 10 mg/kg parenteral dose in the pancreas of rats (Scarpignato et al. 1989; Watanapa et al. 1993), and proglumide was effective against pancreas and hepatocarcinoma at 0.1 mg/ml in drinking water, which meant approximately 30 mg/kg oral dose in mice (Smith et al. 2018; Tucker et al. 2020; Gay et al. 2021). In addition, pre-treatment with CCK antagonists sensitized CFU-GM against carboplatin (Figs. 3 and 4).

Platinum-based drugs have become a mainstay of cancer therapy, approximately half of all patients undergoing chemotherapeutic treatment receive a platinum drug. Carboplatin has some advantages among platina derivatives as it has relatively mild non-hematological toxicities including nephro- and neurotoxicity. In combination with paclitaxel, carboplatin is used for many solid tumors, e.g., ovarian, endometrial, non-small cell lung, and cervical cancers (Gridelli et al. 2018; Egawa-Takata et al. 2022; Ngoi et al. 2022; Liu et al. 2024) and seemed to be successful in treatment of advanced pancreas adenocarcinomas (Chaigneau et al. 2023) and hepatocellular carcinomas (Azmy et al. 2012). The dose-limiting side effect of carboplatin is myelotoxicity with thrombocytopenia and severe neutropenia. In combination, paclitaxel may ameliorate thrombocytopenia but not neutropenia caused by carboplatin (Appleman et al. 2019). The CCK-1R-selective lorglumide pre-treatment resulted in greater increase in sensitivity of CFU-GM and aggravated toxicity of carboplatin more, than the non-selective proglumide (Figs. 3 and 4).

Proglumide, a non-selective CCK receptor antagonist, decreased migration of neutrophils and macrophages into infectious focus in sepsis (Zuelli et al. 2014). Cholecystokinin-2 receptor was determined in human peripheral blood mononuclear cells (Sacerdote et al. 1991; Schmitz et al. 2001) and on polymorphonuclear leukocytes obtained from peripheral blood (Iwata et al. 1996). Both CCK-1R and CCK-2R are found in rat pulmonary interstitial macrophages, and lipopolysaccharide stimulates their gene expressions (Xu et al. 2005). In peritoneal macrophages, CCK-1R and not CCK-2R were detected and gene expression of CCK-1R increased by lipopolysaccharide-stimulation. Anti-inflammatory and immunomodulatory effects of cholecystokinin are realized mostly via CCK-1R, including attenuation of iNOS expression as well as NF kappa B signaling or inhibition of phagocytosis and tissue infiltration with macrophages, which may be useful in sepsis and devazepide, a CCK-1R selective antagonist, inhibiting some of these effects (Saia et al. 2014; Batty et al. 2021).

However, in the HL-60 promyelocytic leukemic cell line, CCK-2R is found (Iwata et al. 1996); there are no data whether they are also found on the normal hemopoietic cells. In our results, we presume that not only CCK-2R, but also CCK-1R are present in normal CFU-GM progenitors and protect them. We were able to detect both CCK-1 and CCK-2 receptors in the CFU-GM enriched bone marrow of LETO rats. Both receptors were downregulated by proglumide using 10 mg/kg orally for 4 weeks. In OLETF rats with CCK-1R deficiency, we detected CCK-2R without CCK-1R (Fig. 6).

We detected both CCK receptors in CFU-GM of LETO rats (Fig. 6), and our results with CCK antagonists suggested that CCK-1R deficiency of obese OLETF rats might contribute at least partly to the increased sensitivity of bone marrow progenitors to carboplatin. It is interesting that the anorexigenic insulin and leptin have a role in maintaining lympho- and myelopoiesis (Claycombe et al. 2008; Machado-Neto et al. 2018), and the protective effect of insulin was proven in granulopoiesis in our previous experiments, too (Benkő et al. 2003). Based on our results, cholecystokinin also may have a protective role in granulopoiesis.

The showed results suggested the role of CCK-1 receptors in granulopoiesis. Therapy failures when leptin or cholecystokinin agonists were tried to suppress appetite in exogenous obesity suggest that not only insulin resistance but also decreased sensitivities against other cytokines may develop in obesity (De la Cruz-Color et al. 2024). It is supported by the findings of Desai et al. (2017) who described that CCK-1 responsiveness varied in the population and was decreased in obese and morbidly obese patients. We found previously that insulin resistance covered progenitors in bone marrow and rosiglitazone, an insulin sensitizer, had a protective effect against myelotoxicity caused by 5-fluorouracil (Benkő et al. 2003; Djazayeri et al. 2005).

Our recent findings supported the results of Miyamoto et al. (2012) about the protective role of cholecystokinin in granulopoiesis via CCK-1R during the production of normal bone marrow-derived macrophages. Cholecystokinin antagonists affected granulopoiesis and sensitized granulocyte–macrophage progenitors against carboplatin toxicity presumably by inhibition of the protective effect of cholecystokinin via the CCK-1 receptor, which is the real specific cholecystokinin receptor. Corresponding with publications about the immunomodulatory effects of cholecystokinin on mature macrophages (Iwata et al. 1996; Xu et al. 2005; Saia et al. 2014; Zuelli et al. 2014), we proved that regulatory functions covered also their progenitors in bone marrow. Further investigations are warranted to understand the function and significance of CCK receptors in granulocyte–macrophage progenitor cells (CFU-GM). If the protective role of cholecystokinin is corroborated in various physiological and pathological processes, it might have value, not only when CCK antagonists are tried for use in malignancies, but also if they are used in other clinical indications in obesity or during immunosuppressive therapies.

## Data Availability

Datasets presented in this article are the property of the University of Debrecen. Requests to access the datasets should be directed to the authors.
